# Ultraviolet B Radiation Triggers DNA Methylation Change and Affects Foraging Behavior of the Clonal Plant *Glechoma longituba*

**DOI:** 10.3389/fpls.2021.633982

**Published:** 2021-02-26

**Authors:** Jiaxin Quan, Vít Latzel, Dan Tie, Yuhan Zhang, Zuzana Münzbergová, Yongfu Chai, Xiao Liu, Ming Yue

**Affiliations:** ^1^Key Laboratory of Resource Biology and Biotechnology in Western China, Ministry of Education, Northwest University, Xi’an, China; ^2^Institute of Botany, Czech Academy of Sciences, Průhonice, Czechia; ^3^Department of Botany, Faculty of Science, Charles University, Prague, Czechia; ^4^Xi’an Botanical Garden of Shaanxi Province/Institute of Botany of Shaanxi Province, Xi’an, China

**Keywords:** clonal plant, habitat selection, heterogeneous environment, foraging behavior, epigenetic memory, UV-B radiation

## Abstract

Clonal plants in heterogeneous environments can benefit from their habitat selection behavior, which enables them to utilize patchily distributed resources efficiently. It has been shown that such behavior can be strongly influenced by their memories on past environmental interactions. Epigenetic variation such as DNA methylation was proposed to be one of the mechanisms involved in the memory. Here, we explored whether the experience with Ultraviolet B (UV-B) radiation triggers epigenetic memory and affects clonal plants’ foraging behavior in an UV-B heterogeneous environment. Parental ramets of *Glechoma longituba* were exposed to UV-B radiation for 15 days or not (controls), and their offspring ramets were allowed to choose light environment enriched with UV-B or not (the species is monopodial and can only choose one environment). Sizes and epigenetic profiles (based on methylation-sensitive amplification polymorphism analysis) of parental and offspring plants from different environments were also analyzed. Parental ramets that have been exposed to UV-B radiation were smaller than ramets from control environment and produced less and smaller offspring ramets. Offspring ramets were placed more often into the control light environment (88.46% ramets) than to the UV-B light environment (11.54% ramets) when parental ramets were exposed to UV-B radiation, which is a manifestation of “escape strategy.” Offspring of control parental ramets show similar preference to the two light environments. Parental ramets exposed to UV-B had lower levels of overall DNA methylation and had different epigenetic profiles than control parental ramets. The methylation of UV-B-stressed parental ramets was maintained among their offspring ramets, although the epigenetic differentiation was reduced after several asexual generations. The parental experience with the UV-B radiation strongly influenced foraging behavior. The memory on the previous environmental interaction enables clonal plants to better interact with a heterogeneous environment and the memory is at least partly based on heritable epigenetic variation.

## Introduction

Essential resources like water, light, and nutrients are usually distributed patchily in natural environments often on scales of a few centimeters (Salzman, 1985; Caldwell and Pearcy, 1994; Stuefer and Hutchings, 1994; Cain et al., 1999; Hutchings and John, 2004; García-palacios et al., 2012). Research shows that, for example, clonal plants can sense the heterogeneity of their microenvironment and make choice between qualitatively different patches by placing ramets to beneficial patches (Bazzaz, 1991; Hutchings and John, 2004; Roiloa and Retuerto, 2012; Oborny and Hubai, 2014; Waters and Watson, 2015). Such foraging behavior likely belongs among the important characteristics enabling the dominance of clonal plants in many ecosystems (Wang et al., 2016; Waters et al., 2016; Dong et al., 2018; Li et al., 2018; Latzel and Münzbergová, 2018; Quan et al., 2018; Chen et al., 2019). Majority of studies explained the microhabitat foraging behavior of clonal plants by morphological plasticity and/or photosynthetic adjustments (Salzman, 1985; Evans and Cain, 1995; Wijesinghe and Hutchings, 1999; Roiloa and Retuerto, 2006a, b, c; Xiao et al., 2006, 2011; Waters and Watson, 2015; Waters et al., 2016; Ye et al., 2015; Quan et al., 2018). However, significantly less is known about the molecular mechanisms that are involved in such behavior (e.g., Latzel and Münzbergová, 2018).

Recently, it has been discovered that the behavior of plants might be altered not only by actual environment but also by previous environmental interactions (Ding et al., 2014; Latzel et al., 2016; Münzbergová and Hadincová, 2017; Latzel and Münzbergová, 2018; Tombesi et al., 2018; Virlouvet et al., 2018). Such memory on past conditions can be enabled by various mechanisms including hormonal signaling or epigenetic changes for example in DNA methylation. Interestingly, clonal plants appear to have greater ability than non-clonal plants to remember past environmental interactions via epigenetic mechanisms (Xiao et al., 2006; Latzel and Klimešová, 2010; Louapre et al., 2012; Douhovnikoff and Dodd, 2015; Dong et al., 2019). This is usually explained by the fact that epigenetic change is easier to be maintained among clonal generations (ramets) than sexual generations due to the lack of meiosis, resetting most of the environmentally induced epigenetic variation (Paszkowski and Grossniklaus, 2011), during clonal growth. It has been thus suggested that epigenetic memory triggered by environmental interactions can serve as an important mechanism contributing to the wide distribution of clonal plants in nature (Latzel and Klimešová, 2010; Verhoeven and Preite, 2014; Douhovnikoff and Dodd, 2015; González et al., 2016; Münzbergová et al., 2019). Ramets produced by clonal growth are potentially independent and genetically identical to the paternal ramet and by definition can be considered as offspring ramets, i.e., next clonal generation (Groenendael et al., 1996). Therefore, the epigenetic memory passed from parental to offspring ramet should be considered as transgenerational (Latzel and Klimešová, 2010; Douhovnikoff and Dodd, 2015).

Epigenetic memory of parental ramets could significantly influence the foraging patterns of offspring ramets and affect thus ultimately habitat selection behavior of the whole genet (Latzel et al., 2016; Latzel and Münzbergová, 2018). Sunlight is undoubtedly the most important environmental factor affecting plant growth. Ultraviolet B (UV-B) radiation (280–315 nm) represents only a small fraction of the solar radiation reaching the Earth’s surface, but has significant impact on plant growth and development. The changes in plant morphology, physiology, and production of secondary metabolites induced by UV-B radiation have been elucidated by a large body of studies (Li et al., 1999; Mackerness, 2000; Frohnmeyer and Staiger, 2003; Vanhaelewyn et al., 2016; Dotto and Casati, 2017).

The radiation intensity of UV-B received by plants is affected by latitude, day time, season, cloud cover, and canopy composition, and plants in nature are thus inevitably exposed to heterogeneous UV-B radiation environment (Liu et al., 2015). Our previous studies had proved that UV-B radiation is one of the most important reasons for low-light-patch distribution of clonal plants under heterogeneous light environment (Zhang et al., 2016). However, the molecular mechanisms behind the habitat selection of clonal plants driven by UV-B radiation are still unknown. We propose that UV-B radiation induces epigenetic changes in exposed parental ramets, which can consequently alter the response of offspring ramets to UV-B patchy environment.

In this study, we explored foraging, growth, and epigenetic response to heterogeneous UV-B environment using a clonal plant, *Glechoma longituba*, and tested the following hypotheses: (1) UV-B experience of parental ramets affects subsequent foraging behavior of the growing clone in heterogeneous UV-B environment; (2) clonal plants can form epigenetic memory of their UV-B experience; and (3) epigenetic memory of UV-B radiation is transmitted among interconnected ramets.

## Materials and Methods

### Plant Material

We used *G. longituba* as the model in the experiment. *G. longituba* is a perennial herb widely distributed in forests, along roadsides or creeks of tropical, subtropical, and temperate areas in China, Vietnam, South Korea, and Russian Far East (Zhang and He, 2009). The species naturally occurs under the canopy, so it experiences environment with heterogeneous UV-B distribution. In the wild, the genus *Glechoma* has two different growth forms. In its flowering phase, from March to June, it produces a vertical stem while it produces plagiotropic, monopodial stolons in vegetative phase. The monopodial stolons are able to creep on the ground and the stolons bear nodes at intervals of 5–10 cm. A pair of opposite, orbicular leaves is produced at each node. Adventitious roots may also develop at the nodes. There is a bud in the axil of each leaf, which may generate a higher-order stolon only when several younger nodes have been produced along the parent stolon (Birch and Hutchings, 1994; Liao et al., 2003). Since the *G. longituba* usually does not flower in the greenhouse, it grew only horizontal stolons in our experimental setting. The *G. longituba* was collected from Jiwozi in Qinling Mountains, Shaanxi, China (33° 47′N, 108°45′ E).

We selected a single genotype of *G. longituba* that was propagated in a greenhouse for 6 months. When we had enough plant material, we severed and replanted 64 youngest ramets (further considered as parental ramets in the study) of comparable size and planted them individually to pots (7 × 7 × 7 cm) filled with soil (25% sand, 25% organic matter, and 50% peat). We allowed the ramets to recover and root for 7 days before we started the main study. The experiment was conducted in a greenhouse from January to March of 2019.

### Design of the Study

**FIGURE 1:**
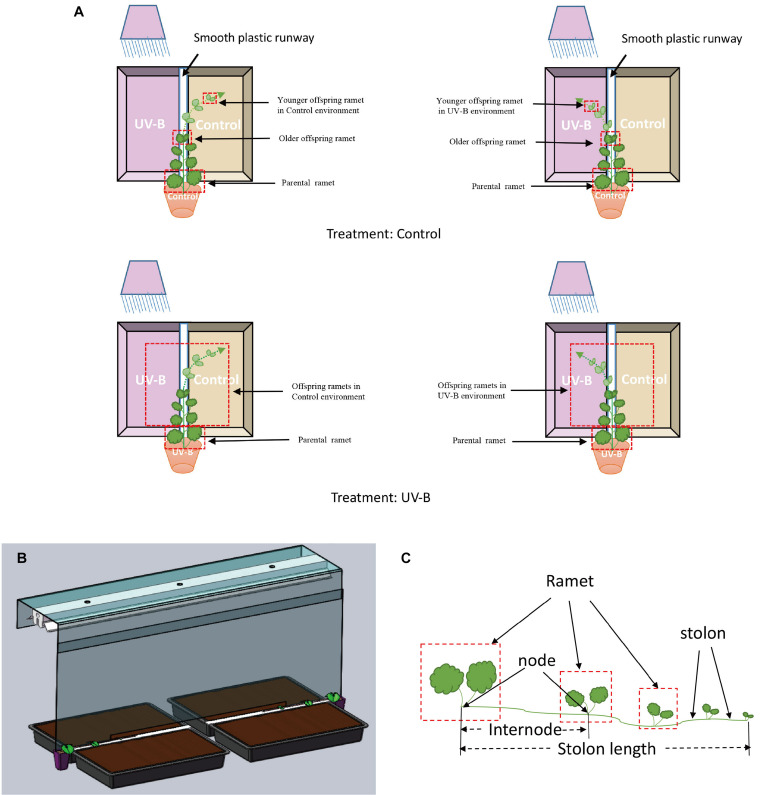
Schema of the experiment. The experiment design consisted of two parental and two offspring light environments: **(A)** Treatment Control: the parental ramet was in control light environment; Treatment UV-B: the parental ramet was in additional 15 day UV-B radiation environment. Then, the newly emerged offspring ramets faced Control light from one side and UV-B radiation from the other side and were free to choose their growth direction (Control pot or UV-B pot, show in dotted lines and arrows). As the plant has only one stolon, each plant can only grow to one of the directions. **(B)** Installation of the experiment. **(C)** Growth pattern of *Glechoma longituba.*

Seven days after parental ramets were planted to pots, we randomly subjected them to two light training environments, Control light environment (further referred to as Control group, *N* = 32), or UV-B radiation for 15 days (further referred to as UV-B group, *N* = 32). The UV-B treatment is described later in the text. After 15 days, the UV-B radiation of the parental ramets was terminated. The newly emerging offspring ramets developing from the parental ramets of both parental light treatments (all parental ramets had single emerging offspring ramet at this point of the study) were allowed to grow in a narrow plastic runway (2 cm wide) for 30 days. Two plastic trays (54 cm long × 28.5 cm wide × 7 cm high) filled with soil (25% and, 25% organic matter, and 50% peat) were placed on the two sides of the runway. The tray on one side of the runway was exposed to Control light environment (Control offspring environment) and the other received additional UV-B radiation (UV-B offspring environment) (Figure 1A). Two parental ramets (one UV-B and one Control) were sharing the same UV-B source (Figure 1B) and thus represented a block. At the beginning, the growth direction of all plant individuals was parallel to the runway, and newly developed interconnected ramets (further referred to as offspring ramets) faced Control light from one side and UV-B radiation from the opposite side. As the growth of the individual continued, it turned its elongating stolon either in one or the other direction, thus selecting the UV-B or Control environment. None of the main stolons remained in the runway—so each main stolon selected one or the other side. In our study, all plants produced only the main stolon but one in which we removed the secondary stolon. To prevent the UV-B radiation penetration to the side of control offspring light environment, a UV-B baffle (0.3 mm transparent polyester film, Dongguan Linuo Plastic Insulation Material Co. LTD, Guangdong, China) was settled between the two types of the light environments (Figure 1). The bottom of the baffle was 2-cm above the plant, ensuring that the newly emerged ramets can sense different light conditions on both sides of the baffle.

During the experiment, the mean irradiance in the greenhouse was 357 ± 20 μmol m^–2^ s^–1^, and humidity was 40 ± 5% with a 14 h/10 h light/dark cycle and a 25/20°C day/night temperature cycle. Plants were watered every 2 days to prevent water stress.

### UV-B Radiation

Following the method of Liu et al. (2015), UV-B radiation was supplied by square-wave UV-B fluorescent lamps (36 W, Beijing Lighting Research Institute, Beijing, China). The maximum output of these lamps was 313 nm. The lamps were wrapped with 0.13 mm cellulose acetate film (Grafix Plastics, Cleveland, OH, transmission down to 290 nm) for the supplemental UV-B radiation group (UV-B group). The lamps were active for 8 h per day from 9:00 to 17:00. The daily radiation dose was 2.88 kJ m^–2^ d^–1^. Unlike the parental ramets that were exposed to UV-B for 15 days, the offspring ramets were exposed to UV-B light radiation for 45 days if they have chosen the UV-B light environment. The amount of UV-B radiation was measured using a UV radiometer (Handy, Beijing, China) every 2 days. The cellulose acetate film was replaced every 5th day.

### Growth and Morphological Parameters

During the experiment, offspring ramets of *G. longituba* grew either into the Control light conditions or into the UV-B light environment (all plants had only the main stolon, so they had only one choice for UV-B or Control environment in our experimental setup). After 45 days (the end of the study), we count the number of offspring selected UV-B or Control environment to calculate the proportion of offspring ramets selection/foraging and measured a range of plant size parameters. First, we recorded biomass, leaf area, and specific leaf area of the parental ramets of every individual, i.e., the part that did not enter the pathway. For the offspring part, we recorded total biomass, total number of offspring ramets, stolon length, total leaf area, and specific leaf area of the whole offspring part of the individual. The biomass includes only above-ground biomass. To assess leaf area, fresh leaves were scanned with a scanner (EPSON Perfection V19, EPSON, China), and leaf area was calculated with Motic software (Motic Images Plus 2.0. Ink, Motic, China). Above-ground biomass was dried for 72 h at 80°C until constant weight and weighted immediately using an electronic balance (SartoriusBT25S, Beijing, China).

### Methylation-Sensitive Amplification Polymorphism (MSAP) Analysis

Methylation alterations in cytosine modification of *G. longituba* were detected using methylation-sensitive amplification polymorphism (MSAP) analysis. For the analyses, we sampled leaves from parental ramet, older offspring (the third offspring ramet counted from the parental ramet on the main stolon, later referred to as older offspring), and the youngest fully developed offspring ramet (the last offspring ramet counted from the parental ramet on the main stolon, later referred to as younger offspring). For each offspring type and combination of parental and offspring environment, we had 3–6 individuals. This unbalanced design was given by the foraging decisions of the plants. The samples were scrubbed gently with 75% ethanol minimize contamination by microorganisms and then dried in silica gel for the subsequent extraction of DNA. This allowed testing whether epigenetic memory of UV-B radiation is transmitted trans-generationally among clonal offspring (ramets). Total genomic DNA was extracted from 30 mg of dry leaves using BioTeKe (Beijing, China), DNA quality was examined by electrophoresis in agarose gel 1% (w/v), and DNA concentration and purity were examined spectrophotometrically with NanoDrop2000 (Thermo Fisher Scientific, United States). The qualified DNA was diluted to the same concentration (100 ng/μl) for MSAP analysis. We used the endonuclease combination 1 μl of *Eco*RI + 1 μl of *Hpa*II (E + H) (NEB, United States) and 1 μl of *Eco*RI + 2 μl of *Msp*I (E + M) (NEB, United States) to double-enzyme genomic DNA, and the digested ends were ligated with 1 μl of *Hpa*II-Msp-adapter (50 pmol/μl), 1 μl of *Eco*RI adapter (5 pmol/μl) (Biotech, China), and 0.5 μl of T4 DNA ligase (TAKARA, Japan). Both the digestion and ligation reactions were performed in a final volume of 20 μl. The enzyme was cut at 37°C for 5 h. Connect at 8°C for 4 h.

The 2 × Taq PCR master mix and pre-amplification and selective amplification primers used in the experiments were synthesized by Shanghai Biotech (Supplementary Appendix Table 1). Both the reactions of pre-amplification and selective amplification were in a final volume of 50 μl. A pre-amplification step was carried out with *Eco*RI pre-amplification primers and *Hpa*II/*Msp*I pre-amplification primers. The PCR mix contained 2 μl of ligated DNA, 21 μl of double-distilled water, 1 μl of H-M pre-amplification primers (10 μM), 1 μl of *Eco*RI pre-amplification primers (10 μM), and 25 μl of 2 × Taq PCR master mix. The pre-amplification conditions were as follows: 72°C for 2 min; 94°C for 2 min; 20 cycles at 94°C for 30 s, 56°C for 30 s, and 72°C for 1 min; and a final elongation step at 72°C for 10 min.

The pre-amplification products were diluted 10 times as a selective amplification template. A selective amplification step was carried out with 11 pairs of selective primer combinations, including the following: *Eco*RI-AAG/*Hpa*II-TGA, *Eco*RI-AAG/ *Hpa*II-TTA, *Eco*RI-AAG/ *Hpa*II-TTG, *Eco*RI-ACT/ *Hpa*II-TCC, *Eco*RI-ACT/ *Hpa*II-TTG, *Eco*RI-AGG/ *Hpa*II-TTC, *Eco*RI-AGG/ *Hpa*II-TGA, *Eco*RI-AGG/ *Hpa*II-TCC, *Eco*RI-AGG/ *Hpa*II-TTG, *Eco*RI-ACG/ *Hpa*II-TTG, and *Eco*RI-AGC/ *Hpa*II-TCC. The PCR mix contained 1 μl of pre-amplified DNA, 22 μl of double-distilled water, 1 μl of H-M selective primer (10 μM), 1 μl of *Eco*RI selective primer (10 μM), and 25 μl of 2 × Taq PCR Master Mix. The selective amplification conditions were as follows: 94°C for 2 min; 10 cycles at 94°C for 30 s; 65°C for 30 s and 72°C for 1 min (each cycle is decremented by 1°C); 23 cycles at 94°C for 30 s; 56°C for 30 s and 72°C for 1 min; and a final elongation step at 72°C for 10 min.

Before polyacrylamide gel, the selective amplification product was inactivated at 70°C for 10 min, then the selective amplification samples were separated by 10% denaturing polyacrylamide gel electrophoresis and subjected to electrophoresis at 220 V for 4 h, and the gel was applied with silver staining. Following staining of the gel, rinsing, developing, and photographing were performed, and band statistical analysis was performed. Fragments from approximately 100–500 bp were scored. The amplified MSAP products were resolved using the method described in Xu et al. (2016).

### Statistical Analyses

#### Growth and Morphological Traits

Because we had too many possibly correlated dependent variables, we used variance inflation factor (VIF) calculated with the “vifstep” function in the R package usdm. We considered variables with VIF values less than 3 as advised by Zuur et al. (2010). While this method has been previously designed to select independent predictors, it can serve the same function when identifying sets of independent response variables. Based on this, we selected two dependent variables (biomass and leaf area) out of three initially measured for the parental plants. For offspring, we selected three dependent variables (offspring ramet biomass, specific leaf area, and ramet number) out of five initially measured. The remaining three dependent variables (specific leaf area of the parental ramet, stolon length, and total leaf area of the whole offspring part) are presented in the Supplementary Appendix Table 2 and Supplementary Appendix Figures 1–3.

One-way ANOVA was used to test the effects of parental training environment (Control vs. UV-B) on biomass and leaf area of parental ramet. Generalized linear model with binomial distribution was used to test the effect of parental environment on habitat selection by the offspring (Control vs. UV-B). Two-way ANOVA was then used to test the effects of parental light environment (Control vs. UV-B) and offspring light environment (Control vs. UV-B) and their interaction on offspring ramets biomass, specific leaf area, and ramet number. Data were transformed when needed (log or square root) to meet the assumptions of homoscedasticity and normality (for details see Table 1). Ramet number followed Poisson distribution. The effects of paternal and offspring environment and their interaction on these variables were tested using generalized linear model with the respective distribution. All analyses were conducted using R 3.5.1. Initially, we used block as a covariate in our models. As its inclusion did not affect the results, we present results without its inclusion.

**TABLE 1:** ANOVA results for effects of parental environment (Control vs. UV-B) and offspring environment (Control vs. UV-B) on morphological traits of offspring ramet of *Glechoma longituba*.

	Offspring biomass^b^		Specific leaf area ^a^		Ramet number	
	*F*_(1_, _53)_	*P*	*F*_1_, _52_	*P*	*D*_1_, _56_	Pr(> Chi)
Parental (Pa)	**59.71**	** < 0.001**	**6.45**	**0.014**	2.02	0.155
Offspring (Off)	0.69	0.407	2.08	0.155	0.02	0.892
Pa × Off	2.08	0.156	2.29	0.136	0.86	0.354

*Degrees of freedom (df) and F and P-values are given. Values for P < 0.05 are in bold. ^a^log transformation. ^b^sqrt transformation.*

#### DNA Methylation Variation

From the fragment presence/absence score matrix of both enzymatic reactions, the methylated state of every locus (5′-CCGG target) was assessed: presence of both *Eco*RI–*Hpa*II and *Eco*RI–*Msp*I products (1/1) denotes an unmethylated state, presence of only one of the *Eco*RI–*Hpa*II (1/0) or *Eco*RI–*Msp*I (0/1) products represents methylated states (hemi-methylated or internal methylation), and absence of both *Eco*RI–*Hpa*II and *Eco*RI–*Msp*I products (0/0) was considered as an uninformative state (Salmon et al., 2008; Pérez Figueroa, 2013; Wang et al., 2019). We used the “vegan” package of R (Dixon, 2003) to calculate Shannon’s diversity index of each individual based on these data. Methylation level (%) was calculated by dividing MSAP bands representing methylated 5′-CCGG sites (differential presence/absence of restricted fragments in *Hpa*II and *Msp*I assays) against the total number of scored bands (Liu et al., 2012).

The binary matrix of methylated state (Loci composition) was analyzed by canonical correspondence analysis (CCA) with function capscale in the “vegan” package of R (Dixon, 2003; Bonin et al., 2007). The epigenetic diversity (Shannon’s diversity index) and methylation level were tested using a generalized linear model. In all cases, we first tested the effect of parental environment (Control and UV-B), ramet age, and their interaction using the whole data, i.e., parental (Control and UV-B) and older and younger offspring ramets. Afterward, we tested the effect of parental environment, offspring environment, ramet age (old/young), and their interactions using only data from the offspring. In both cases, we accounted for the fact that the ramets of different age belong to the same individual. We did this by using the parent identity as a random factor and the individual code as random factor in the univariate analyses and by defining the individual as a hierarchical level in the multivariate analysis. Due to significant interactions with ramet age, we also repeated the tests separately for the ramets of different ages. All analyses were conducted using R 3.5.1.

## Results

**FIGURE 2:**
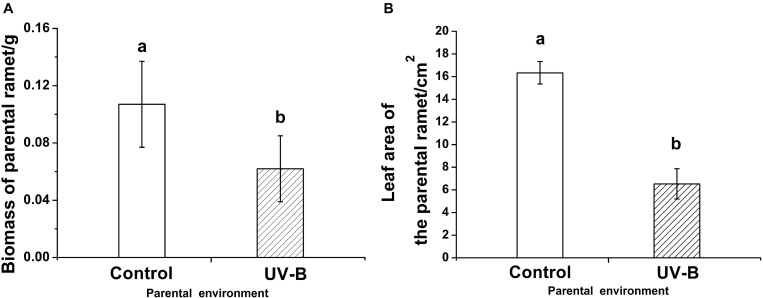
The biomass (*F* = 17.95, *P* < 0.001) **(A)** and the leaf area (*F* = 36.68, *P* < 0.001) **(B)** of the parental ramet in different parental light environment. Control: parental ramet in control light environment, UV-B: parental ramet experienced 15 day UV-B radiation. The graphs show mean ± SE. Columns sharing the same letter are not significantly different from each other at *P* < 0.05.

The parental biomass (*F* = 17.95, *P* < 0.001) and parental leaf area (*F* = 36.68, *P* < 0.001) of *G. longituba* were both significantly lower under UV-B environment than under Control environment (Figure 2).

**FIGURE 3:**
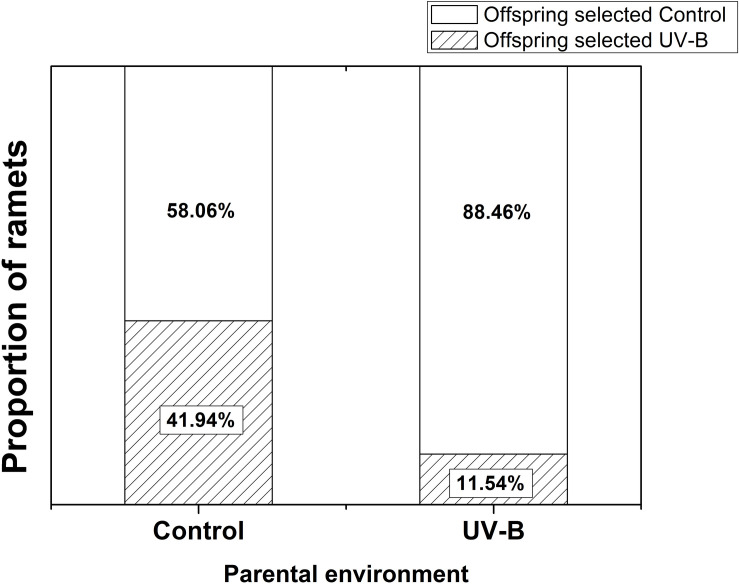
Proportional selection for different offspring light environments by offspring ramets. Control: parental ramet trained in control light environment, UV-B: parental ramet trained in UV-B light environment (*n* = 32 per parental treatment; Control vs. UV-B: Residual Deviance = 6.91; *P* = 0.009).

**FIGURE 4:**
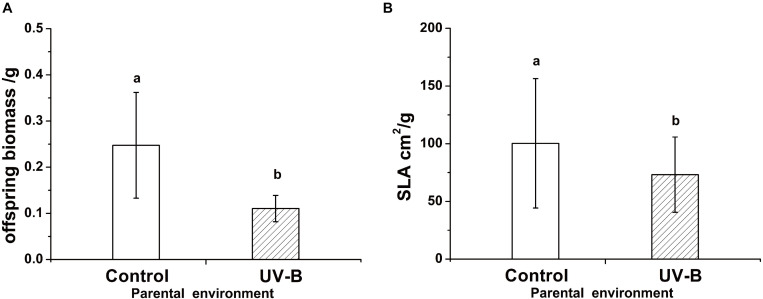
The offspring biomass (*F* = 59.71, *P* < 0.001) **(A)** and specific leaf area (*F* = 6.45, *P* < 0.001) **(B)** in different parental light environment. Control: parental ramet in control light environment, UV-B: parental ramet in UV-B radiation. The graphs show mean ± SE. Columns sharing the same letter are not significantly different from each other at *P* < 0.05.

Foraging for different light environment was significantly affected by the training light environment previously experienced by the parental ramet (Residual Deviance = 6.91; *P* = 0.009). Plants of Control group (i.e., control training light environment) placed 58% offspring ramets in Control offspring light environment whereas the proportion increased to 88.46% in plants developed from parental ramets trained in UV-B (UV-B group, Figure 3). The offspring biomass and specific leaf area were significantly higher for offspring of parents trained in Control parental light than in UV-B parental light environment, while the ramet number was unaffected by parental training conditions (Table 1 and Figure 4). There was no significant effect of offspring light environment or interaction between parental and offspring environment in any of the variables (Table 1).

### MSAP Analyses

A total of 105 MSAP loci were amplified from each individual using 11 primer pair combinations. When MSAP profiles of all ramet types were analyzed together (i.e., parental and older and younger offspring ramet), loci composition, epigenetic diversity (Shannon’s diversity index), and overall DNA methylation level were significantly affected by parental training environment (Control vs. UV-B, Table 2, Pa). Shannon’s diversity index (*I*_epi_) and overall DNA methylation level (*L*_epi_) were significantly lower for ramets of UV-B trained parents (*I*_epi_ = 3.82 ± 0.22; *L*_epi_ = 44.34 ± 0.09%) than Control trained parents (*I*_epi_ = 3.97 ± 0.14; *L*_epi_ = 50.86 ± 0.07%). Loci composition, but not diversity and methylation level, was significantly affected by ramet type. Loci composition significantly differed between parental training environment and ramet type (Table 2, Pa × Ramet). Therefore, we consequently tested the effects of parental and offspring environment on each ramet type separately.

**TABLE 2:** ANOVA/CCA analyses results for effects of parental environment (Control vs. UV-B), and all ramet type (parental ramet, older offspring ramet, and younger offspring ramet) on loci composition and epigenetic diversity (Shannon’s diversity index) and total DNA methylation level of *Glechoma longituba*.

		Loci composition		Shannon’s diversity index		DNA methylation level	
	df	*F*	*P*	*F*	*P*	*F*	*P*
Parental environment (Pa)	1.50	**2.14**	**0.038**	**7.15**	**0.010**	**6.75**	**0.012**
Ramet type (Ramet)	2.34	**2.41**	**0.002**	1.76	0.182	1.81	0.174
Pa × Ramet	2.48	**1.65**	**0.004**	0.61	0.549	0.65	0.528

*Degrees of freedom (df) and F and P-values are given. Values for P < 0.05 are in bold.*

In case of the parental ramet, the loci composition (*F* = 1.81; *P* = 0.003; df = 1.16), Shannon’s diversity index (Control = 4.00 ± 0.07, UV-B = 3.70 ± 0.25; *F* = 11.78; *P* = 0.003), and total DNA methylation level (Control = 52.28 ± 0.04%, UV-B = 39.58 ± 0.10%; *F* = 13.32; *P* = 0.002) significantly depended on the parental training environment.

When analyzing offspring ramets (older offspring ramet and younger offspring ramet), we found only loci composition to be significantly affected by parental training environment (Table 3). Offspring environment had no effect on any of the variables.

**TABLE 3:** ANOVA/CCA results for effects of parental environment (Control vs. UV-B), offspring environment (Control vs. UV-B) on loci composition, epigenetic diversity (Shannon’s diversity index), and total DNA methylation level of offspring ramet (older and younger offspring ramet) of *Glechoma longituba*.

		Loci composition		Shannon’s diversity index		DNA methylation level	
	df	*F*	*P*	*F*	*P*	*F*	*P*
**Older offspring ramet**							
Parental environment (Pa)	1.15	**1.46**	**0.032**	0.22	0.643	0.25	0.622
Offspring environment (Off)	1.15	1.12	0.281	0.13	0.724	0.07	0.793
Pa × Off	1.14	1.01	0.411	3.40	0.086	3.50	0.083
**Younger offspring ramet**							
Parental environment (Pa)	1.15	1.55	0.052	1.30	0.274	1.36	0.264
Offspring environment (Off)	1.15	0.83	0.723	0.36	0.560	0.25	0.627
Pa × Off	1.14	0.91	0.631	0.31	0.589	0.47	0.506

*Degrees of freedom (df) and F and P-values are given. Values for P < 0.05 are in bold.*

## Discussion

### Training of Parental Ramet to UV-B Radiation Affects Foraging Behavior of a Genet

We demonstrate that the 15-day long training of parental ramets of *G. longituba* to increased UV-B radiation has a negative effect on their growth (biomass) as well as on the number and biomass of offspring ramets and hence on the fitness of the whole individual. This is in line with several other studies (Frohnmeyer and Staiger, 2003; Kakani et al., 2003; Vanhaelewyn et al., 2016; Dotto and Casati, 2017). However, our study provides an additional unique finding that the experience of parental ramet with UV-B radiation strongly affects consequent foraging behavior for light of the growing individual. Individuals with the parental ramet’s experience with UV-B radiation preferentially placed offspring ramets to light conditions without UV-B radiation in comparison to the genetically identical individuals without the parental experience with UV-B radiation (see Figure 3). Such behavior probably helped to mitigate negative consequences of UV-B radiation on the individual’s fitness. Avoiding patches with high UV-B levels for already weakened (offspring ramets of UV-B stressed parental ramet) individuals may reduce further negative impact of UV-B radiation on fitness of the genets. Such a behavior can be considered as an escape strategy, which has been also documented in other clonal species (de Kroon and Hutchings, 1995; Ye et al., 2006; Ikegami et al., 2007; Puijalon et al., 2008).

Our study adds to the mounting evidence that the behavior of clonal plants is not independent on their environmental interactions in the past. For example, Louapre et al. (2012) showed that the foraging behavior of clonal plants *Potentilla reptans* and *Potentilla anserina* is affected by the nutrient availability in older ramets. Latzel and Münzbergová (2018) found that clonal plant *Fragaria vesca* is able to store information on the light and nutrient availability of older ramets and based on this information decide where to place offspring ramets, which they consider as an exhibition of anticipatory behavior in clonal plants (Latzel and Münzbergová, 2018). A puzzling question is which mechanisms allow for the memory on the past environmental interactions and consequent change in foraging behavior of clonal plants.

### Mechanisms Allowing for the Change in Foraging Behavior

It is very likely that the information passed from parental to interconnected offspring ramets is enabled by their connection via stolons. Some researchers suggest that the connection between ramets allows not only for transport of water with dissolved assimilates but also for transmission of signaling molecules like phytohormones (e.g., Alpert and Mooney, 1986; Hutchings, 1999; Stuefer et al., 2004; Gómez et al., 2008; Louapre et al., 2012; Waters and Watson, 2015). Hence, parental ramet can communicate with offspring ramets (and vice versa), which can alter overall behavior of the genet. Another theory suggests that it is shared epigenetic memory that can be involved in the behavior of clonal plants (González et al., 2016, 2018; Latzel et al., 2016). It has been reported that memories on the environmental interactions can be stored and transmitted to next generations via epigenetic change such as the change in DNA methylation (Molinier et al., 2006; Bossdorf et al., 2008; Boyko et al., 2010; Verhoeven et al., 2010; González et al., 2016; Latzel et al., 2016; Richards et al., 2017). Latzel et al. (2016) suggested that parental ramet can carry epigenetic information about its experiences with environmental interactions and pass the information to its offspring ramets. Hence, behavior of clonal plants can be strongly influenced by epigenetic memories on the past environments (Latzel et al., 2016).

In this study, we demonstrated that UV-B radiation significantly reduced DNA methylation level and Shannon’s diversity index of parental ramets. Similar reduction in DNA methylation due to increased UV radiation was reported also for *Zea mays* (Steward, 2002; Sokolova et al., 2014), *Picea abies* (Ohlsson et al., 2013), and *Artemisia annua* (Pandey and Pandey-Rai, 2015; Pandey et al., 2019). Moreover, the MSAP analysis revealed that the loci composition of parental ramets that experienced UV-B radiation significantly differed from parental ramets subjected to control light conditions. Both results suggest that DNA methylation change was involved in response to the UV-B stress. Our study also provides some evidence that the UV-B-induced DNA methylation variation can be, to some degree, passed to connected clonal offspring ramets and involved in the change of foraging behavior. Different loci composition triggered by the parental UV-B treatment was detected in both older and younger offspring ramets. In addition, the observed reduced level of DNA methylation of parental ramets was inherited by older but not younger offspring ramets (Table 3). A similar pattern was detected for Shannon’s diversity index. This suggests that the epigenetic memory can be passed from parental to offspring ramets through mitotic cell division, but is gradually degrading during clonal growth (i.e., after several asexual generations, in our case, the younger ramet was usually the fourth clonal generation derived from the older offspring ramet). A similar conclusion was reached by Shi and colleagues (Shi et al., 2019) on a clonal plant alligator weed (*Alternanthera philoxeroides)*. They found that environmentally induced epigenetic variation is gradually resetting when plants of different populations (environments) are transplanted to a common garden. After 10 asexual generations and 2 years of cultivation in a common garden, plants of previously different epigenetic profiles become epigenetically comparable (Shi et al., 2019). Our findings thus suggest that the foraging behavior of clonal plants might be at least partly under epigenetic control, which supports the model of epigenetically coordinated advanced behavior of clonal plants described by Latzel et al. (2016). However, better insights into the role of epigenetic memory in the observed changes in foraging behavior require more sophisticated molecular methods such as whole genome bisulfite sequencing (Richards et al., 2017).

## Conclusion

Our results demonstrated that the experience of parental ramet with UV-B radiation can affect foraging behavior of the clonal plant in an UV-B heterogeneous environment. Genets with UV-B-stressed parent adopted “escape strategy” in a heterogeneous environment by avoiding an environment with UV-B radiation and by plastic change in leaf area, stolon length, and ramets number. These results point out the importance of information sharing among parent–offspring ramets that can strongly influence behavior of clonal plants with significant impact on their overall fitness. Hence, it is evident that the behavior of clonal plants can be highly sophisticated, combining the interaction of actual environmental conditions and/or environmental heterogeneity with information from the past. Such cross-talks between actual and past experiences might provide clonal plants considerable advantage in their “understanding” of the environment. Our study also suggests that epigenetic memory can play a role in the observed change in behavior; nonetheless, more studies that employ sophisticated molecular analyses, e.g., NGS, are needed to provide unambiguous evidence.

## Data Availability Statement

The original contributions presented in the study are included in the article/Supplementary Material, further inquiries can be directed to the corresponding author/s.

## Author Contributions

XL and JQ conceived and designed the experiments. JQ performed the experiments. ZM helped with the data analysis. JQ and VL wrote the manuscript and others provided editorial advice. All authors read and approved the final manuscript.

## Conflict of Interest

The authors declare that the research was conducted in the absence of any commercial or financial relationships that could be construed as a potential conflict of interest.
