# Supplementary material for: Ultraviolet B Radiation Triggers DNA Methylation Change and Affects Foraging Behavior of the Clonal Plant Glechoma longituba
Source: Front Plant Sci. 2021 Feb 26;12:633982. doi: 10.3389/fpls.2021.633982 (PMC7952652; doi:10.3389/fpls.2021.633982)
Supplement: Supplementary file 1 [file Table_1.DOCX]

Appendix Table 1 Sequences of adapters and primers used for Methylation-Sensitive Amplification Polymorphism analysis

| Adapters/primers | *Eco*RI | *Hpa*II/*Msp*I |
| --- | --- | --- |
| Adapter | 5'-CTCGTAGACTGCGTACC-3' | 5'-GATCATGAGTCCTGCT-3' |
|  | 5'-AATTGGTACGCAGTC-3' | 5'-CGAGCAGGACTCATGA-3' |
| Pre-amplification primers | 5'-GACTGCGTACCAATTCA-3' | 5'-ATCATGAGTCCTGCTCGGT-3' |
| Selective primers | 5'-GACTGCGTACCAATTC ACT-3' | 5'-ATCATGAGTCCTGCTCGG TCC-3' |
|  | 5'-GACTGCGTACCAATTC AGG-3' | 5'-ATCATGAGTCCTGCTCGG TTG-3' |
|  | 5'-GACTGCGTACCAATTC ACG-3' | 5'-ATCATGAGTCCTGCTCGG TTC-3' |
|  | 5'- GACTGCGTACCAATTC AAG-3' | 5'-ATCATGAGTCCTGCTCGG TGA-3' |
|  | 5'-GACTGCGTACCAATTC AGC-3' | 5'-ATCATGAGTCCTGCTCGG TTA-3' |

Appendix Table 2 ANOVA results for effects of parental environment (Control *versus* UV-B) and offspring environment (Control *versus* UV-B) on morphological traits of offspring ramet of *Glechoma longituba*.

|  | leaf area^a^ | | | stolon length | |  |
| --- | --- | --- | --- | --- | --- | --- |
|  | F _1,53_ | | P | F _1,52_ | P |  |
| Parental (Pa) | **75.75** | | **<0.001** | **44.10** | **<0.001** | |
| Offspring (Off) | **8.91** | | **0.004** | 0.23 | 0.630 | |
| Pa × Off | 0.003 | 0.955 | | 0.16 | 0.694 | |

Degrees of freedom (df), F and P values are given. Values for P < 0.05 are in bold. alog transformation.
