# Supplementary material for: Ultraviolet B Radiation Triggers DNA Methylation Change and Affects Foraging Behavior of the Clonal Plant Glechoma longituba
Source: Front Plant Sci. 2021 Feb 26;12:633982. doi: 10.3389/fpls.2021.633982 (PMC7952652; doi:10.3389/fpls.2021.633982)
Supplement: Supplementary file 2 [file Table_2.DOCX]

Appendix Figure 1 The specific leaf area (F = 13.78, P < 0.001) of parental ramets in different parental light environment. Control: parental ramet in control light environment, UV-B: parental ramet experienced 15-d UV-B radiation. The graphs show mean ± SE. Columns sharing the same letter are not significantly different from each other at p < 0.05.

Appendix Figure 2 The stolon length (F = 44.10, P < 0.001) of offspring in different parental light environment. Control: parental ramet in control light environment, UV-B: parental ramet in UV-B radiation. The graphs show mean ± SE. Columns sharing the same letter are not significantly different from each other at p < 0.05.

Appendix Figure 3 The leaf area of (F = 75.75, P < 0.001) offspring in different parental light environment. Control: parental ramet in control light environment, UV-B: parental ramet in UV-B radiation. The graphs show mean ± SE. Columns sharing the same letter are not significantly different from each other at p < 0.05.
